# The “SALPARE study” of spontaneous intracerebral haemorrhage—part 2-early CT predictors of outcome in ICH: keeping it simple

**DOI:** 10.1186/s42466-022-00228-2

**Published:** 2023-01-12

**Authors:** Renzo Manara, Ludovica De Rosa, Francesca Vodret, Caterina Kulyk, Renato Pennella, Eleonora Contrino, Giacomo Cester, Francesco Causin, Alessio Pieroni, Federica Viaro, Maria Luisa Zedde, Rosario Pascarella, Rosa Napoletano, Claudio Baracchini

**Affiliations:** 1 Neuroradiology Unit, Padua University Hospital, Padua, Italy; 2 Stroke Unit and Neurosonology Laboratory, Padua University Hospital, Padua, Italy; 3 Stroke Unit and Neurosonology Laboratory, Department of Neurology, Johannes Kepler University Linz, Linz, Austria; 4 Neuroradiology, Department of Medicine and Surgery, University of Salerno, Salerno, Italy; 5 Neurology Unit, Stroke Unit, AUSL-IRCCS di Reggio Emilia, Reggio Emilia, Italy; 6 Neuroradiology Unit, Azienda Unità Sanitaria Locale-IRCCS di Reggio Emilia, Reggio Emilia, Italy; 7 UOC Neurologia AOU S. Giovanni di Dio e Ruggi d’Aragona, Salerno, Italy

**Keywords:** Cerebral hemorrhage, Hematoma expansion, NCCT markers

## Abstract

**Background:**

The aim of this study was to investigate the prognostic role of hematoma characteristics and hematoma expansion (HE) in patients with spontaneous intracerebral hemorrhage (ICH).

**Methods:**

This multicenter prospective cohort study enrolled consecutive adult patients with non-traumatic ICH admitted to three Italian academic hospitals (Salerno, Padova, Reggio Emilia) over a 2-year period. Early noncontrast CT (NCCT) features of the hematoma, including markers of HE, and 3-month outcome were recorded. Multivariable logistic regression analysis was performed to identify predictors of poor outcome.

**Results:**

A total of 682 patients were included in the study [mean age: 73 ± 14 years; 316 (46.3%) females]. Pontine and massive hemorrhage, intraventricular bleeding, baseline hematoma volume > 15 mL, blend sign, swirl sign, margin irregularity ≥ 4, density heterogeneity ≥ 3, hypodensity ≥ 1, island sign, satellite sign, and black hole sign were associated with a higher risk of mortality and disability. However, at multivariate analysis only initial hematoma volume (OR 29.71) proved to be an independent predictor of poor functional outcome at 3 months.

**Conclusion:**

Simple hematoma volume measured on baseline CT best identifies patients with a worse outcome, while early NCCT markers of HE do not seem to add any clinically significant information.

## Introduction

The search for early radiological predictors of clinical outcome has been of the utmost importance since the introduction of brain CT in the diagnostic work-up of ICH. Several studies have shown that hematoma expansion (HE) is a valuable predictor of poor outcome [1–3] and a potentially important therapeutic target. However, HE can be detected only in the second phase of the disease, when some patients have already died or it is too late for specific medical and/or surgical procedures. In recent years, several authors have proposed neuroradiological hematoma features of shape and density as predictors of HE to possibly select patients who might benefit from a more aggressive treatment [4, 5]. These markers have the advantage of being evaluated in an emergency room setting by non-contrast CT (NCCT) without advanced neuroimaging, but their use remains unclear as outcome predictors in large ICH studies. Therefore we decided to conduct a multicenter prospective study in three academic centers representing Northern, Central and Southern Italy in order to investigate the prognostic role of hematoma characteristics and HE in patients with ICH.

## Patients and methods

### Study population

This prospective multicenter cohort study enrolled consecutive adult consecutive adult (≥ 18 years) patients with non-traumatic ICH admitted to three Italian University Hospitals (Padova, Reggio Emilia and Salerno) over a 2-year period (January 2016–December 2017). Recruitment criteria are presented in part I.

### Data collection

Demographic characteristics, vascular risk profile, clinical data and radiological characteristics were recorded as described in Part I. The degree of disability before stroke was based on an anamnestic modified Rankin Scale (mRS 0–5), while functional outcome at 90 days was assessed at the 3-month follow-up visit (mRS 0–5) or anamnestically for the deceased patients (mRS 6).

The types of multidetector CT were Siemens 16 slices in Salerno, Philips 64 slices in Padova, and Philips 128 slices in Reggio Emilia. Furthermore, slice thickness was < 4 mm.

Concerning baseline NCCT, the following characteristics were recorded: ICH location (deep, lobar, infratentorial), hematoma volume, intraventricular extension, and markers of HE. The hematoma volume was calculated according to the ABC/2 method, where A is the greatest hemorrhage diameter, B is the diameter 90 degrees to A, and C is the approximate number of CT slices with hemorrhage multiplied by the slice thickness [6, 7]. In particular, massive hemorrhage is defined as a lesion with at least 3 cm in largest dimension in the cerebral hemispheres or 1.5 cm in the brain stem [8].

Several NCCT markers have been proposed to assess greater risk of hematoma expansion: blend sign, swirl sign, intra-hematoma hypodensities, irregular hematoma shape, heterogeneous hematoma density, island sign, satellite sign and black hole sign. The last three markers were evaluated only in the group of patients recruited at the University hospital of Padova. Briefly, blend sign is defined as a hypoattenuating area next to a hyperattenuating area of the hematoma, with sharp separation between the two regions and a density difference of at least 18 Hounsfield units [9]. Swirl sign is defined as a region or regions of hypoattenuation or isoattenuation (compared to the attenuation of brain parenchyma) within the ICH. The areas of hypoattenuation or isoattenuation may vary in shape and should be observed on both axial and coronal plane [10]. Intra-hematoma hypodensities are defined as any hypodense region (compared with the surrounding acute blood) encapsulated within the hemorrhage and lacking any connection with the brain parenchyma around the bleeding. We distinguished four types of hypodensities based on the distinctness of their margins and their relative density, as previously described by Boulouis et al. [11]. Hematoma density and shape were rated with an ordinal scale ranging from 0 to 5 (the score increases with higher irregularity in shape and heterogeneous hematoma density), as previously described by Barras et al. [12]. Island sign is defined as ≥ 3 scattered small hematomas all separate from the main hematoma (separated islands) or ≥ 4 small hematomas some or all of which may connect with the main hematoma (connected islands) [13]. Satellite sign is defined as scattered high-density lesions completely separate from the main hemorrhage in at least the single axial slice [14]. Black hole sign is defined as any hypodense region (compared with the surrounding acute blood) encapsulated within the hemorrhage, presenting a clear border and a density difference of at least 28 Hounsfield units by comparison to the adjacent hemorrhage [15].

Early CTA performed within 6 h from ICH onset, and brain MRI were included in the study analysis whenever available.

As per study protocol, patients undergoing surgery (superficial hemorrhage; clot volume between 20 and 80 ml; worsening neurological status; hemorrhage causing midline shift/raised ICP) were excluded from the final analysis.

Data on time interval between first and second CT as well as HE (relative hematoma volume growth > 33% or absolute hematoma volume growth > 6 mL) [2] were also recorded.

The independent committee for the evaluation of initial and follow-up CT scans, early CTA (within 6 h from onset) and cerebral MRI images was composed by 3 radiologists: A.S. in Salerno, R.P. in Reggio Emilia, and F.C. in Padova. All images were centrally evaluated by R.M., a neuroradiologist with more than 20-year experience in cerebrovascular diseases and blind to the patient’s clinical picture.

Data collection was approved by the Ethics Committee of each hospital and informed consent was obtained from participants or their family members.

Patients were treated medically or surgically at the physician’s discretion, according to the standard of care of each recruiting center. In particular, patients treated with surgery were selected before the second CT, based on the size and the location of the hemorrhage at onset, and according to the judgment of the neurosurgeon in charge. Those who died or underwent intracranial surgery before follow-up CT scan were excluded from the evaluation of the hematoma expansion. Notably, patients without CT signs of neoplasia or arteriovenous malformation were included even if this diagnosis was achieved during the follow-up, since the aim of the study was to identify outcome predictors in presumably spontaneous brain hemorrhages regardless of the final diagnosis.

### Statistical analysis

All statistical analyses were performed using SPSS Version 26 and STATISTICA Version 13.

Continuous variables were presented using means or medians; categorical variables were presented as percentages.

Baseline radiological characteristics of ICH and CT markers of hematoma expansion were included as candidate variables. Patients’ outcome at 90 days was categorized as favorable (mRS ≤ 2) or poor (mRS ≥ 3). Statistical significance was assessed by Student *t* test, Mann–Whitney *U*-test and chi-square, as appropriate. The test characteristics (odds ratio, sensitivity, specificity and 95% confidence intervals) were calculated directly for disconnected variables or after dichotomization (Youden method) for ordinal and normal variables. A *p* < 0.05 was considered significant. Multivariate analysis was performed using the ordinal logistic regression model to identify the independent predictors of functional outcome after 3 months. The ordinal variable was obtained by stratifying the outcome as class 0 (mRS 0–2), class 1 (mRS 3–5) and class 2 (mRS 6).

## Results

Baseline radiological cohort characteristics are shown in Table 1. Among 727 hematomas observed, about half (348; 47.8%) was located in the cerebral lobes, just over a quarter (201; 27.1%) in the striatal/capsular area, about one-sixth (107; 14.7%) in the thalamus and/or thalamo-capsular area, and 3.9% (28) in the brainstem. The distribution of hematoma location and baseline hematoma volume was not significantly different among patients of the three academic hospitals.

**Table 1:** Baseline radiological cohort characteristics

	All centers (n = 727)	Padova (n = 336)	Salerno (n = 194)	Reggio Emilia (n = 197)
*Location*				
Cerebral lobes	348 (48%)	163 (48.5%)	85 (44%)	100 (51%)
Striato-capsular area				
Anterior	25 (3.4%)	12 (3.6%)	7 (3.6%)	6 (3.1%)
Middle	7 (1%)	5 (1.5%)	2 (1%)	0
Lateral	40 (5.5%)	17 (5.1%)	9 (4.6%)	14 (7.2%)
Posteromedial	8 (1.1%)	1 (0.3%)	4 (2.1%)	3 (1.6%)
Posterolateral	56 (7.7%)	29 (8.6%)	13 (6.7%)	14 (7.2%)
Massive	65 (8.9%)	26 (7.7%)	26 (13.4%)	13 (6.7%)
Thalamocapsular area	59 (8%)	30 (8.9%)	14 (7.2%)	15 (7.7%)
Thalamus	48 (6.6%)	22 (6.6%)	13 (6.7%)	13 (6.7%)
Cerebellum	42 (5.8%)	25 (7.4%)	10 (5.1%)	7 (3.6%)
Pons	24 (3.3%)	5 (1.5%)	9 (4.6%)	10 (5.1%)
Midbrain	4 (0.6%)	1 (0.3%)	1 (0.5%)	2 (0.1%)
Undetectable	1 (0.1%)	0	1 (0.5%)	0
*Volume (ml, mean ± SD)*	40.6 ± 53.1	39.9 ± 53.5	39.4 ± 51.6	41.6 ± 53.9
*Intraventricular bleeding*	335 (46%)	166 (49.4%)	86 (44.3%)	83 (42.1%)
*Intrahematoma hypodensities*				
0	347 (47.7%)	134 (39.9%)	107 (55.2%)	106 (53.8%)
1	93 (12.8%)	47 (14%)	21 (10.8%)	25 (12.7%)
2	168 (23.1%)	90 (26.8%)	36 (18.6%)	42 (21.3%)
3	23 (3.2%)	16 (4.7%)	3 (1.5%)	4 (2%)
4	96 (13.2%)	49 (14.6%)	27 (13.9%)	20 (10.2%)
*Blend sign*	145 (20%)	80 (23.8%)	32 (16.5%)	33 (16.8%)
*Swirl sign*	417 (57.4%)	203 (60.4%)	111 (57.2%)	103 (52.3%)
*Irregular hematoma shape*				
1	146 (20.1%)	53 (15.8%)	47 (24.2%)	46 (23.3%)
2	92 (12.6%)	49 (14.6%)	22 (11.3%)	21 (10.7%)
3	93 (12.8%)	40 (11.9%)	24 (12.4%)	29 (14.7%)
4	43 (5.9%)	24 (7.1%)	8 (4.1%)	11 (5.6%)
5	353 (48.6%)	170 (50.6%)	93 (48%)	90 (45.7%)
*Heterogeneous hematoma density*				
1	218 (30%)	84 (25%)	66 (34%)	68 (34.5%)
2	127 (17.5%)	63 (18.7%)	25 (12.9%)	39 (19.8%)
3	99 (13.7%)	49 (14.6%)	21 (10.8%)	29 (14.7%)
4	62 (8.5%)	32 (9.5%)	17 (8.8%)	13 (6.6%)
5	220 (30.3%)	108 (32.2%)	64 (33%)	48 (24.4%)
*Island sign*^a^	108 (32.1%)			
*Satellite sign*^a^	202 (60.1%)			
*Black hole sign*^a^	86 (25.6%)			

^a^Assessed only in the Padua cohort (n = 336)

Follow-up CT was obtained in 556 patients (81.5%), with no significant differences among recruiting centers (Padova: 81.5%; Reggio Emilia: 82%; Salerno: 80.6%). However, time-to-follow-up CT differed significantly (31 ± 25 h in Padova, 64 ± 61 h in Salerno and 51 ± 77 h in Reggio Emilia; *p* < 0.001). Control CT was performed within 24 h in 388 patients (55%), namely 225 (67%) in Padua, 64 (35%) in Salerno, and 99 (53%) in Reggio Emilia (*p* < 0.001).

The most frequently observed marker of HE was the satellite sign (60.1%) while the least frequent was the blend sign (20%). HE occurred in 164/556 patients (29.5%) and did not differ among centers (70/261, 26.8% in Padova; 45/145, 31.0% in Salerno and 49/150, 32.7% in Reggio Emilia; *p* < 0.05).

### Outcome predictors

Pontine and massive hemorrhage, intraventricular bleeding, larger baseline hematoma volume (> 15 mL), blend sign, swirl sign, high margin irregularity (score ≥ 4), high density heterogeneity (score ≥ 3), one or more hypodensities, island sign, satellite sign, and black hole sign were associated with a higher risk of poor functional outcome (Table 2).

**Table 2:** Univariate and multivariate regression analyses for predictors of poor outcome (mRS > 2)

Variables	Univariate		Multivariate	
	*P* value	OR	*P* value	OR
Pontine or massive hemorrhage^a^	0.01374	1.24 (1.09–2.75)	NS	
Intraventricular bleeding	0.00000	3.94 (2.52–6.16)	NS	
Baseline hematoma volume > 15 ml	0.00000	6.67 (4.15–10.72)	0.00000	29.71
Hematoma expansion (volume growth > 33% or > 6 ml)	0.00087	2.42 (1.42–4.11)	NS	
Blend sign	0.00694	4.20 (2.76–6.39)	NS	
Swirl sign	0.00000	4.20 (2.76–6.39)	NS	
Margin irregularity ≥ 4	0.00000	3.85 (2.52–5.86)	NS	
Density heterogeneity ≥ 3	0.00000	3.75 (2.45–5.72)	NS	
Hypodensity ≥ 1	0.00000	2.70 (1.80–4.05)	NS	
Island sign^b^	0.00030	3.73 (1.76–7.91)	NS	
Satellite sign^b^	0.00642	2.14 (1.23–3.73)	NS	
Black hole sign^b^	0.02912	2.21 (1.07–4.60)	NS	

^a^Massive hemorrhage: lesions at least 3 cm in largest dimension in the cerebral hemispheres or 1.5 cm in the brain stem

^b^Assessed only in the Padua cohort (n = 336)

However, in a multivariable logistic regression model that included clinical characteristics (see Part I), the only CT feature that was found to be independently associated with a poor outcome was a baseline hematoma volume > 15 ml (Table 2).

## Discussion

This prospective multicenter study on spontaneous ICH has shown that pontine location and early NCCT are highly predictive of 3-month poor outcome and mortality. However, among several hematoma features only baseline hematoma volume proved to be reliable in stratifying patients’ risk.

Classically, the most frequent site of ICH has been the internal capsule and the striatum, often related to chronic arterial hypertension [16, 17]. Interestingly, this study has shown a new epidemiological trend, as lobar ICH is the leading type of cerebral hemorrhage in all three recruiting centers. Lobar hemorrhages are often the distinguishing feature of amyloid angiopathy, a degenerative disease thought to be related to alleles of the apolipoprotein E gene, allowing for increased amyloid deposition within vessel walls [18]. Prognosis is worse and the recurrence rate is higher [19]. Yet, in our study a lobar location was not associated with a worse clinical outcome, meaning that other conditions such as arterial hypertension triggered a lobar ICH.

In recent years, different NCCT markers have been shown to be associated with HE [20, 21]. Specifically, shape-related features seem to identify hematomas in an intermediate stage of maturity, with peripheral sites of secondary bleeding and increased intra hemorrhage pressure [10, 12–14, 22]. Other non-contrast markers include heterogeneous hematoma density, which might reflect active bleeding or impaired coagulation processes [9, 11, 12, 15, 20]. These neuroradiological signs may represent a reliable and easy-to-use alternative to the spot sign, since CTA is not widely available and in many centers is not routinely performed in the acute phase of ICH (see Part I). However, some reports have highlighted several limits: a relevant overlap between different signs, a low intra- and inter-rater reliability, a time-consuming technique, and a required expertise in acute neuroimaging of ICH [23–26].

Early NCCT signs have been repeatedly proven to predict HE [27–30]. As HE is strongly related to an unfavourable outcome, the detection of these early neuroradiological features has been suggested as an indicator for starting treatment aimed at prevention of hematoma growth. Nonetheless, it is not clear which signs or combination of signs best identify patients at high risk of ICH expansion.

Several recent studies have indeed tested early NCCT markers as predictors of outcome [13, 31–38] (Table 3). Yet, most of them were retrospective single-center cohort studies with relatively small sample sizes, or secondary analysis of randomized clinical trials with strict inclusion criteria that might misrepresent the whole ICH population. Our multicenter prospective study enrolled consecutive spontaneous ICH patients regardless of hematoma size or location, anticoagulation treatment, CT timing, and concomitant early CTA. In addition, it included a multivariate analysis encompassing relevant confounders such as initial hematoma volume, CTA spot sign, ongoing antithrombotic treatment, comorbidities, and clinical severity.

**Table 3:** Major studies on NCCT markers of hematoma expansion

Study	Design and inclusion period	Sample size	Enrollment criteria	Outcome definition	Multivariate analysis	Results
Selariu 2012	Retrospective single-center [2007–2009]	203	All patients with ICD code I10.1-9	3-month mRS 4–6 and 30 days mortality	Yes	Swirl sign was independently associated with death/poor outcome after ICH
Delcourt 2016	Secondary analysis of multicenter RCT (INTERACT2) [2008–2012]	2066	Excluded if: massive hematoma with poor prognosis; GCS < 5; antihypertensive treatment initiated > 6 h after symptom onset	3-month mRS 3–6	Yes	Irregular shape, but not heterogeneous density, was independently associated with poor outcome after ICH
Boulouis 2016	Retrospective analysis of prospective single-center cohort [1994–2015]	800	Excluded if: volume < 1 cc; two or more simultaneous ICHs	3-month mRS 4–6	Yes	Hypodensities were independently associated with poor outcome
Li Q 2017	Prospective single-center [2011–2016]	252	Excluded if: anticoagulant-associated ICH	3-month mRS 4–6	Yes	Island sign was independently associated with poor outcome
Morotti 2017	Secondary analysis of multicenter RCT (ATACHII) [2010–2015]	952	Excluded if: GCS < 5; volume > 60 cc; antihypertensive treatment initiated > 4.5 h after symptom onset	3-month mRS 4–6	Yes	Hypodensities, blend sign, irregular shape and heterogeneous density were independently associated with poor outcome. Yet, there was no evidence of an interaction between CT markers and benefit from intensive BP reduction
Sporns 2018	Retrospective multicenter [2013–2017]	201	Excluded if: CTA not performed or performed > 6 h after symptom onset; anticoagulant-associated ICH	Discharge mRS 4–6	Yes	Hypodensities and swirl sign were independently associated with poor outcome
Law 2019	Secondary analysis of multicenter RCT (TICH-2) [2013–2017]	2077	Excluded if: admitted to hospital > 8 h after symptom onset; anticoagulant- associated ICH; mRS > 4; life expectancy < 3 months; GCS < 5; contraindication to tranexamic acid	3-month mRS 4–6	Yes	Black hole sign, hypodensities and island sign were independently associated with poor outcome. Yet, CT markers did not predict a better response to tranexamic acid
Quintas-Neves 2019	Retrospective single-center [2014–2017]	328	Excluded if: acquired or hereditary coagulation diseases; CT performed > 24 h after symptom onset	30 days mortality	Yes	Only irregular margins and satellite sign were independently associated with mortality, but they have suboptimal diagnostic test performances for such outcome
Our study 2022	Prospective multicenter [2016–2017]	682	All spontaneous ICH	3-month mRS 4–6 and mortality	Yes	Only baseline volume was independently associated with poor outcome and mortality

*RCT* randomized clinical trial, *ICH* intracerebral hemorrhage, *mRS* modified Rankin Scale, *GCS* Glasgow Coma Scale

In our population, early CT markers were all associated with a poor outcome after ICH. Nonetheless, at multivariate analysis only baseline hematoma volume proved to be an independent predictor of disability and mortality. Our hypothesis for this finding is that large hematomas are more prone to present shape- and density- related features that have been shown to correlate with a worse outcome. Indeed, a larger size of the hematoma at onset likely results from several factors including the size of the vessel involved, the impairment of the coagulation system, specific features of the tissue surrounding the hematoma such as leukoaraiosis, the site of hematoma, the compression of intracranial vessel leading to secondary ischemia or increased venous pressure, etc. Most of the CT markers of hematoma expansion likely represent features linked to rebleeding (swirl sign, hematoma in-homogeneity, etc.). Moreover, some of these characteristics might be reasonably related to preceding anticoagulation therapy, even though our multivariate analysis did not confirm this treatment as an independent predictor of poor outcome (see Part I).

Therefore, according to the present study, hematoma evaluation might be strikingly simplified and focused on hematoma size thus making CT a more user-friendly tool even in the emergency room of less specialized centers that are indeed the first point of care of most ICH patients.

## Conclusion

Hematoma volume > 15 ml on baseline CT best identifies patients with a worse outcome while early NCCT markers of hematoma expansion do not seem to add any clinically significant information.

## Data Availability

All data generated or analysed during this study are included in this published article.
